# Recovery of phosphorus as soluble phosphates from aqueous solutions using chitosan hydrogel sorbents

**DOI:** 10.1038/s41598-021-96416-2

**Published:** 2021-08-18

**Authors:** Tomasz Jóźwiak, Agata Kowalkowska, Urszula Filipkowska, Joanna Struk-Sokołowska, Ludmila Bolozan, Luminita Gache, Marius Ilie

**Affiliations:** 1 Department of Environmental Engineering, University of Warmia and Mazury in Olsztyn, Warszawska St. 117a, 10-957 Olsztyn, Poland; 2 Department of Environmental Engineering Technology, Bialystok University of Technology, Wiejska St. 45E, 15-351 Bialystok, Poland; 3 Faculty of Chemical Engineering and Environmental Protection, Gheorghe Asachi Technical University of Iaşi, Bulevardul Profesor Dimitrie Mangeron 67, 700050 Iaşi, Romania

**Keywords:** Environmental chemistry, Green chemistry, Polymer chemistry

## Abstract

This manuscript presents new method of phosphorus recovery from aqueous solutions in a convenient form of readily-soluble phosphates using chitosan hydrogels. Non-modified chitosan hydrogel granules (CHs) and chitosan hydrogel granules crosslinked with epichlorohydrin (CHs-ECH) served as orthophosphate ion carriers. The developed method was based on cyclic sorption/desorption of orthophosphates, with desorption performed in each cycle to the same solution (the concentrate). The concentrations of orthophosphates obtained in the concentrates depended on, i.a., sorbent type, sorption pH, source solution concentration, and desorption pH. Phosphorus concentrations in the concentrates were even 30 times higher than these in the source solutions. The maximum concentrate concentrations reached 332.0 mg P-PO_4_/L for CHs and 971.6 mg P-PO_4_/L for CHs-ECH. The experimental series with CHs-ECH were characterized by higher concentrations of the obtained concentrate, however the concentrates were also more contaminated with Cl^−^ and Na^+^ ions compared to series with CHs. The high content of chlorine and sodium ions in the concentrates was also favored by the low pH of sorption (pH < 4) and very high pH of desorption (pH > 12) in the cycles. After concentrate evaporation, phosphorus content in the sediment ranged from 17.81 to 19.83% for CHs and from 16.04 to 17.74% for CHs-ECH.

## Introduction

Phosphorus is an element of vast economic importance. It is not only essential for each living organism’s vital functions but also an integral and irreplaceable component of fertilizers and feedstuffs, and also an important constituent of detergents. Sources of phosphorus exploitation for the global economy’s needs include its non-renewable, natural deposits in the form of apatites and phosphorites^1^. Continuous growth of the global population entails, among other things, the increased production of foods and cleansing agents. The growing industry’s demand for phosphorus compounds accelerates the exploitation of its depleting deposits. With the current phosphorus deposits exploitation rate, they are estimated that economically feasible phosphate rock will be exhausted within 100–400 years^2,3^. The deficit of this raw material will firstly affect European Union Member States whose natural phosphorus resources are scarce or of no economic importance^4^. Therefore, searching alternative phosphorus sources seems to be a priority, emphasizing its recovery and recycling. Today, the treatment of high-phosphorus wastewater appears to offer greater potential for the recovery of this nutrient^5–8^.

Several methods for phosphorus recovery directly from wastewater have been developed so far^9,10^. The simplest of these include the precipitation methods, and the most often used precipitating agents are calcium compounds^11^. The sludge formed upon precipitation is rich in calcium phosphates to be used as a raw material for the production of mineral fertilizers^12^, which inscribes into the phosphorus recycling strategy. However, this method’s drawbacks include the necessity of using a precipitating agent, wastewater salinity, and a relatively low process efficiency.

The nanofiltration method is also a well-known mean to recover phosphorus from wastewater. It does not require strong chemicals and can be used even for wastewater containing low concentrations of phosphorus^13^. This method also requires the presence of calcium ions in the wastewater, which must be introduced to the system, e.g., in the form of calcium hydroxide. The wastewater is pumped under high pressure through a semi-permeable polyamide membrane, which traps calcium phosphate microcrystals. The crystals accumulated on the membrane are then dissolved in the acid solution to obtain a concentrated solution of orthophosphates. The disadvantages of this method include very high investment and operating costs.

Phosphorus can also be recovered from wastewater via the chemical precipitation of magnesium-ammonium phosphate hexahydrate, i.e., struvite^14^. Struvite can be directly used in agriculture as a substitute for phosphorus-based mineral fertilizers^15^. However, the possibilities of phosphorus recovery in the form of struvite are often limited due to specific technical requirements and the difficulty of controlling it. There is also the possibility of biological struvite precipitation using dedicated bacteria, like e.g., *Myxococcus xanthus*^16^, which allow producing its crystals. In turn, the use of *Brevibacterium antiquum* results in the formation of magnesium phosphate crystals^17^. Currently, this process is effective only under strictly controlled laboratory conditions.

Newer methods for phosphorus recovery from wastewater include these based on the crystallization of hydroxyapatite under alkaline conditions (DHV Crystalactor process, KURITA). Patented technologies, however, are expensive, require complicated installation, and strictly defined process parameters^18–20^.

The literature provides information on attempts to recover phosphorus from aqueous solutions using sorbents^21,22^. Many research works with "phosphorus recovery" in their title actually focus only on phosphorus sorption and possibly sorbent regeneration, omitting analyses of the actual nutrient recovery^23–26^. In published works, which describe the actual process of phosphorus recovery from aqueous solutions, sorbents were used as carriers for orthophosphate ions, which were transported from the source solution to the target (desorption) solution during sorption/desorption. After desorption, the phosphate ions were precipitated with calcium compounds, e.g., CaCl_2_ or Ca(OH)_2_, for their easier separation from the solution. Phosphorus recovered in this way was usually in the form of a water-insoluble sediment containing calcium phosphate—Ca_3_(PO_4_)_2_. The sorbent could be regenerated after each desorption and reused^27,28^. The most frequently tested sorbents for phosphorus recovery from aqueous solutions include mineral materials such as metal oxides and hydroxides^21,22,29^ as well as "Layered double hydroxides"—ion exchangers^30,31^. A major drawback of the sorption-based phosphorus recovery methods is the relatively low sorption efficiency of the tested materials, which translates into a low process efficiency. A bigger problem, however, is usually posed by the insoluble form of the recovered phosphorus (phosphate of a divalent or trivalent metal). Reuse of phosphorus contained in calcium phosphate requires its conversion into a soluble form, e.g., by its reaction with hydrochloric acid^32^. This, however, makes the phosphorus recycling process considerably more complicated.

This work presents the feasibility of efficient phosphorus recovery from aqueous solutions in the form of convenient and readily-soluble phosphate salts.

### Concept of the novel method for phosphorus recovery from aqueous solutions

**Figure 1:**
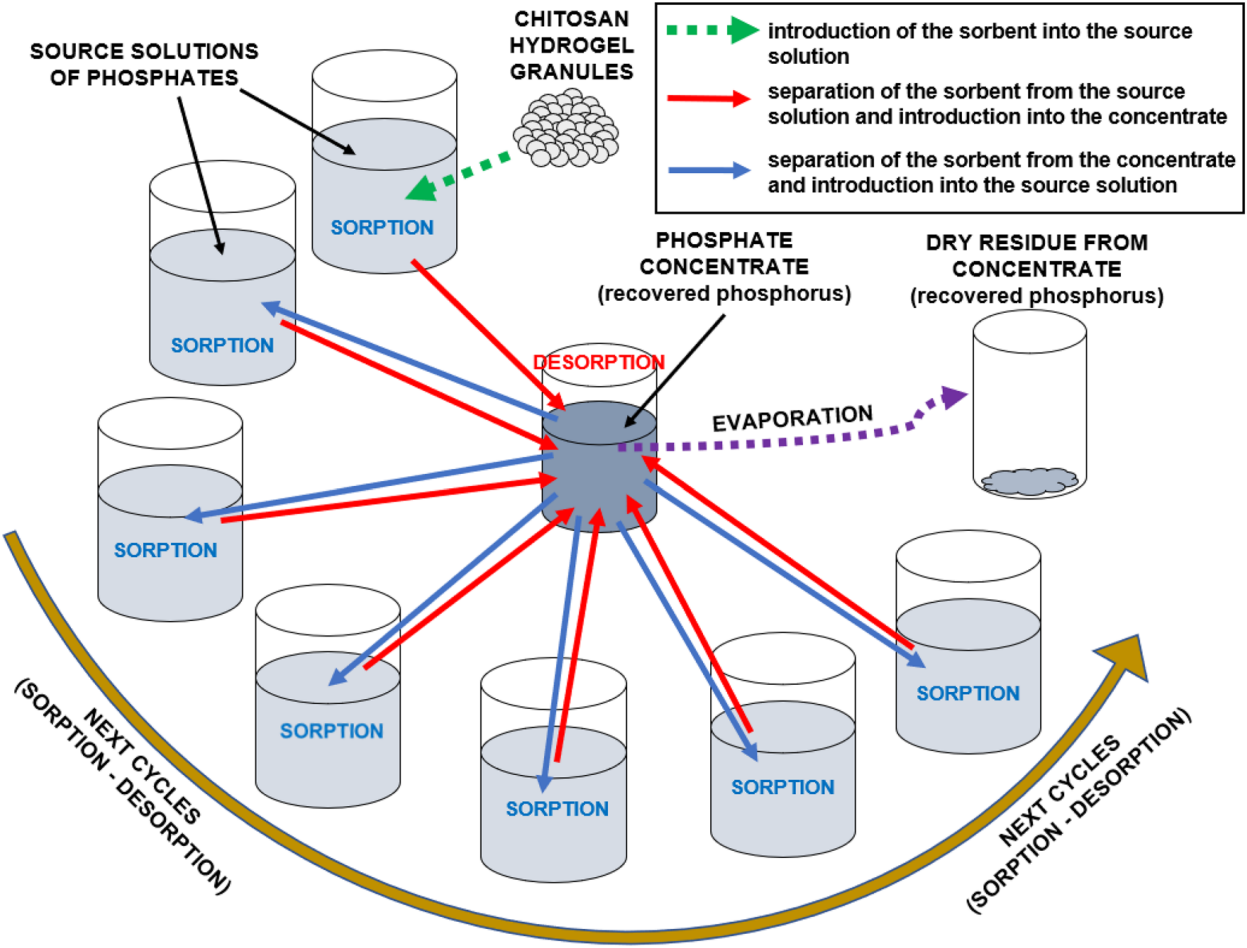
General scheme of the presented method for phosphorus recovery from aqueous solutions in the form of a concentrate and a dry residue from the concentrate.

The presented method focuses on recovering phosphorus in the form of orthophosphate ions from the source solution, due to its dominance in municipal sewage. The process is based on cyclic sorption/desorption of orthophosphates, following the principle of "sorption in a large volume and desorption in a small volume of wastewater”. In each cycle, the desorption is performed into the same solution, leading to the production of a "concentrate". Its concentration increases with each successive cycle until the maximum value. All sorption/desorption cycles are carried out with one and the same sorbent batch. The sorbent is not regenerated between cycles. The obtained "concentrate" can be evaporated, producing sediment with a high phosphorus content (Fig. 1). Since no phosphorus precipitants are needed in this process, the recovered phosphorus has a convenient, water-soluble form of monovalent light metal phosphates.

In the discussed process, hydrogel chitosan granules are used as carriers of orthophosphate ions. Chitosan is a polysaccharide, a deacetylated form of chitin. On an industrial scale, it is obtained from the exoskeletons of sea crustaceans. The possibility of extracting chitosan from the waste products of the seafood processing industry makes it a widely available and relatively cheap material. A characteristic feature of chitosan is its basic character caused by the presence of –NH_2_ groups in its structure. Chitosan based sorbents already proved their good sorption properties in relation to heavy metals^33–37^ and dyes^38^. In this study, the choice of chitosan as the base material for sorbent production was driven by the proven high affinity of orthophosphate ions to its amine functional groups. The sorption capacity of a properly prepared chitosan sorbent may even exceed 400 mg PO_4_^3−^/g. The hydrogel form of chitosan improves the penetration of orthophosphate ions into the sorption centers located inside the sorbent and also facilitates quick separation of the sorbent from the solution. The crosslinked form of a hydrogel (with epichlorohydrin) used in some research ensured its stability at pH 3, which is recommended during orthophosphate sorption^39^. Since the binding of orthophosphate ions onto chitosan proceeds via the electrostatic interaction between the sorbate and the sorbent functional groups, the process can theoretically be easily controlled by pH correction.

The most important results, which are also indicators of the method’s efficiency, are the maximum concentration of phosphorus in the concentrate as well as its percentage content in the dry residue. The efficiency of sorption and desorption in individual cycles is essential as well. Presumably, the course of phosphorus recovery from aqueous solutions depends mainly on the composition of source solutions and on the conditions of the sorption and desorption processes.

This study investigated the impact of such parameters as: concentration of orthophosphates in source solutions, pH of sorption and desorption, as well as sorbent type on the sorption/desorption efficiency in subsequent cycles, the maximum concentration of the phosphate "concentrate" obtained, and phosphorus content in the dry residue left after concentrate evaporation. It was conducted with chitosan hydrogel granules crosslinked with epichlorohydrin [CHs-ECH] and unmodified chitosan hydrogel granules [CHs] used as carriers of orthophosphate ions.

## Materials

### Chitosan—raw material for sorbent production

Chitosan used in the study was in the form of flakes (2–3 mm) (Heppe Medical Chitosan GmbH, Halle, Germany). It was produced from chitin derived from shrimp exoskeletons and characterized by a medium deacetylation degree of DD = 85.0% (DD = 82.6–87.5%) and viscosity of 500 mPa (1% wt. in 1% acetic acid, temp. 25 °C). Its molecular weight ranged from 50 to 190 kDa. The total content of heavy metals (Pb, Hg, Cd) in the polymer did not exceed 25 ppm.

### Chemical reagents

The following reagents were used in the study:

- epichlorohydrin (99%) (ACROS ORGANICS, Poland)—for crosslinking chitosan gel
- hydrochloric acid (37%) (POCH S.A., Poland)—for pH correction in aqueous solutions
- sodium hydroxide (> 98%) (POCH S.A., Poland)—for hydrogel gelling and pH correction in aqueous solutions
- acetic acid (> 99%) (POCH S.A., Poland)—for dissolving chitosan flakes
- sodium dihydrogen phosphate 1 monohydrate (NaH_2_PO_4_ × 1 H_2_O), (> 99%) (POCH S.A., Poland)—for preparing stock and source solutions.

## Methods

### Preparation of non-crosslinked chitosan hydrogel granules (CHs)

25 g d.m. of chitosan flakes, 925 g of water, and 50 g of acetic acid were added into a beaker (vol. 2000 mL) and stirred with a spatula until chitosan dissolved completely. The resulting solution with a chitosan concentration of 2.5% was left for 12 h to deaerate. Next, it was instilled, using a 0.8 × 40 injection needle, to 2 M NaOH to form hydrogel granules (2.0–2.2 mm in diameter). Once formed, the hydrogel granules were kept in 2 M NaOH for 24 h, and afterward rinsed with deionized water on a laboratory screen until neutral pH of the filtrate. The ready sorbent in the form of chitosan hydrogel granules (CHs) was stored in deionized water having a temperature of 4 °C. The dry mass content in CHs was 5.4%.

### Preparation of chitosan hydrogel granules crosslinked with epichlorohydrin (CHs-ECH)

The non-modified chitosan hydrogel granules (CHs) were weighed (10 g d.m.) into a conical flask (300 mL). Then, an epichlorohydrin (ECH) solution (100 mL, concentration of 3.2 g/L, pH 11) was added to the flask. The sorbent was prepared with a minimal dose of epichlorohydrin (0.032 g ECH/g_CHs_) ensuring complete stability of the chitosan hydrogel in a wide range of pH values, i.e., pH 1–12^40^. The flask was protected with a parafilm and placed for 24 h in a water bath with a shaker (Water bath shaker type 357, Elpin-Plus, Poland; 150 r.p.m., oscillation amplitude 20 mm, 60 °C). Afterward, the crosslinked chitosan was sifted on a laboratory screen and rinsed with deionized water until neutral pH of the filtrate. The resulting hydrogel sorbent crosslinked with epichlorohydrin (CHs-ECH) was stored in deionized water (temp. 4 °C). The dry mass content in CHs-ECH was 4.2%.

FTIR analysis of the hydrogel sorbents: CHs and CHs-ECH, indicating the presence of –NH_2_ groups (crucial for phosphorus binding), is presented in Supplement 1.

### Analysis of pH effect on orthophosphate sorption efficiency

The hydrogel sorbent (0.1 g d.m.) was weighed into a series of conical flasks (250 mL), to which solutions of sodium dihydrogen phosphate (NaH_2_PO_4_) were added (100 mL, 10 mg P-PO_4_/L, pH 2–12). Next, the flasks were placed on a multi-station shaker (Basic Shaker SK-71, JEIO TECH, Korea), and shaken at 150 r.p.m. and oscillation amplitude of 32 mm. After 2 h, samples (10 mL) were collected from the solution to determine the concentration of P-PO_4_ left in the solutions. Solutions’ pH was measured after sorption as well. Experiments were performed in triplicate.

### Analysis of orthophosphate sorption kinetics

The chitosan sorbent (2.0 g d.m.) was weighed into a series of flasks (2500 mL). Then, NaH_2_PO_4_ solutions (2000 mL, 10/100 mg P-PO_4_/L) with an optimal sorption pH (established in point 3.3) were poured into the flasks. The flasks were placed on a magnetic stirrer (150 r.p.m.) (Multi-Channel Stirrer MS-53 M, JEIO TECH, Korea). In specified time intervals (after: 0, 5, 10, 15, 20, 30, 45, 60, 90, 120, 150, 180, and 240 min), samples (5 mL) were collected from the flasks using an automatic pipette to determine P-PO_4_ concentration in the solution. Experiments were performed in triplicate.

### Analysis of pH effect on orthophosphate desorption efficiency

The chitosan hydrogel sorbents were pre-treated by orthophosphate sorption using the NaH_2_PO_4_ solution (10 mg P-PO_4_/L) with pH value optimal for each sorbent (established in point 3.3). The sorbent was used in a dose of 1 g/L. The sorption process was conducted on a multi-station shaker (150 r.p.m.). The contact time of the sorbent with the sorption solution was established in point 3.4.

The hydrogel sorbents with sorbed orthophosphates were sifted on a screen and weighed (in 0.1 g d.m. portions) into empty conical flasks (250 mL). Next, NaH_2_PO_4_ solutions (100 mL) with pH 7–12 (deionized water with pH corrected using NaOH solutions) were poured into the flasks, which were then placed on a shaker (150 r.p.m., oscillation amplitude 32 mm). After 2 h, samples (10 mL) were collected from each flask to determine the concentration of P-PO_4_ in desorption solutions. Solutions’ pH was measured as well. Experiments were performed in triplicate.

### Analysis of orthophosphate desorption kinetics

As in point 3.4, the hydrogel sorbent wase pre-treated by orthophosphate sorption (at concentrations of 10/100 mg P-PO_4_/L) at the optimal sorption pH (established in point 3.3) and optimal contact time of the sorbent with the solution (established in point 3.4).

The sorbents prepared were weighed (in 2.0 g d.m. portions) into beakers (2500 mL). Next, NaOH solutions (2000 mL) with pH 12/pH 13 were added, and the beakers were placed on magnetic stirrers (150 r.p.m.). After 0, 5, 10, 15, 20, 30, 45, 60, 90, 120, 150, 180, and 240 min, samples (5 mL) were collected from the flasks to determine P-PO_4_ concentration in desorption solutions. Experiments were performed in triplicate.

### Analysis of the efficiency of orthophosphate sorption/desorption cycles

The chitosan sorbents were weighed (in 5.0 g d.m. portions) into a series of flasks (5000 mL). Then, NaH_2_PO_4_ solutions with concentrations of 10/50/100 mg P-PO_4_/L and an optimal sorption pH (established for each sorbent in point 3.3) were poured into the flasks. The flasks were then placed on a magnetic stirrer (150 r.p.m) for the optimal period of the sorption process (established in point 3.4). Afterward, the sorbents were separated from source solutions using laboratory screens. Then, samples (100 mL) of the source solutions were collected to determine P-PO_4_ concentration (later on, also pH and TDS were measured in these samples). The sifted sorbents were transferred to wide beakers (1000 mL). Then, NaOH solutions with pH 12/pH 13 were added to the beakers, which were next placed on a multi-station magnetic stirrer (150 r.p.m.). After optimal desorption time (established in point 3.6), the sorbent was separated from the desorption solution (concentrate) using laboratory screens. Samples (2 mL) were collected from the concentrate to determine P-PO_4_ concentration. After completed desorption, also pH and TDS were measured in the concentrate.

The sorbent’s batch separated from the concentrate in the first sorption/desorption cycle was used in the successive cycles. In the subsequent cycles, the sorption process was always performed in newly-prepared source solutions, whereas the desorption process was always conducted to the same desorption solution (concentrate). In each cycle, before the sorbent was added to the concentrate, its pH was corrected (to pH 12/pH 13) using small amounts of 5 M NaOH, to ensure that desorption would proceed at the same pH in the successive cycles. Between the cycles, the sorbents were neither rinsed nor regenerated in any way. The successive cycles were continued till the sorbents lost their capability for orthophosphate ions uptake from the source solutions (till concentrations of source solutions and concentrate were no longer changing). In total, 12 experimental series were conducted, differing in the concentration of the source solution (10/50/100 mg P-PO_4_/L), pH of desorption solution (concentrate) (pH 12/pH 13), and sorbent type (CHs/CHs-ECH).

After the last effective cycle, analyses were conducted for the content of chlorides in the phosphate concentrate and for the dry residue of the concentrate, following Polish Standard PN-78/C-04541 (gravimetric method).

### Physicochemical analyses

The content of orthophosphates was determined using the spectrophotometric method with ammonium molybdate (PN-EN ISO 6878:2006). The spectrophotometric analysis was carried out using a UV-3100 PC spectrophotometer (VWR Spectrophotometers, Canada). TDS and pH were determined in the source solutions, and the phosphate concentrate using a PL-700 ALS multifunctional tester (GOnDO Electronic, Taiwan). The content of chlorides in selected samples was determined using cuvette tests by HACH.

### Computation methods

The amount of phosphorus bound by the chitosan sorbents was computed from the formula:

1 $$ {\text{Q}} = \left( {{\text{C}}_{{{\text{start}}}} - {\text{C}}_{{\text{E}}} } \right) \times {\text{V}}/{\text{m}} $$

where Q—amount of bound phosphorus [mg P-PO_4_/g]; C_start—_phosphorus concentration before sorption [mg P-PO_4_/L]; C_E—_phosphorus concentration after sorption [mg P-PO_4_/L]; V—solution volume [L], m—sorbent mass [g d.m.].

Phosphorus sorption and desorption kinetics were described using the pseudo-first order Eq. (2) and the pseudo-second order Eq. (3):

2 $$ {\text{dq}}/{\text{dt}} = {\text{k}}_{1} \times \left( {{\text{q}}_{{\text{e}}} - {\text{q}}} \right) $$

3 $$ {\text{dq}}/{\text{dt}} = {\text{k}}_{2} \times ({\text{q}}_{{\text{e}}} - {\text{q}})^{2} $$

where k_1—_constant in the pseudo-first order equation [L/min], k_2—_constant in the pseudo-second order equation [L/min], q_e—_equilibrium amount of sorbed/desorbed phosphorus [mg P-PO_4_/g]; q—momentary amount of sorbed/desorbed phosphorus [mg P-PO_4_/g].

## Results and discussion

### Effect of pH on i-PO4 sorption and desorption

**Figure 2:**
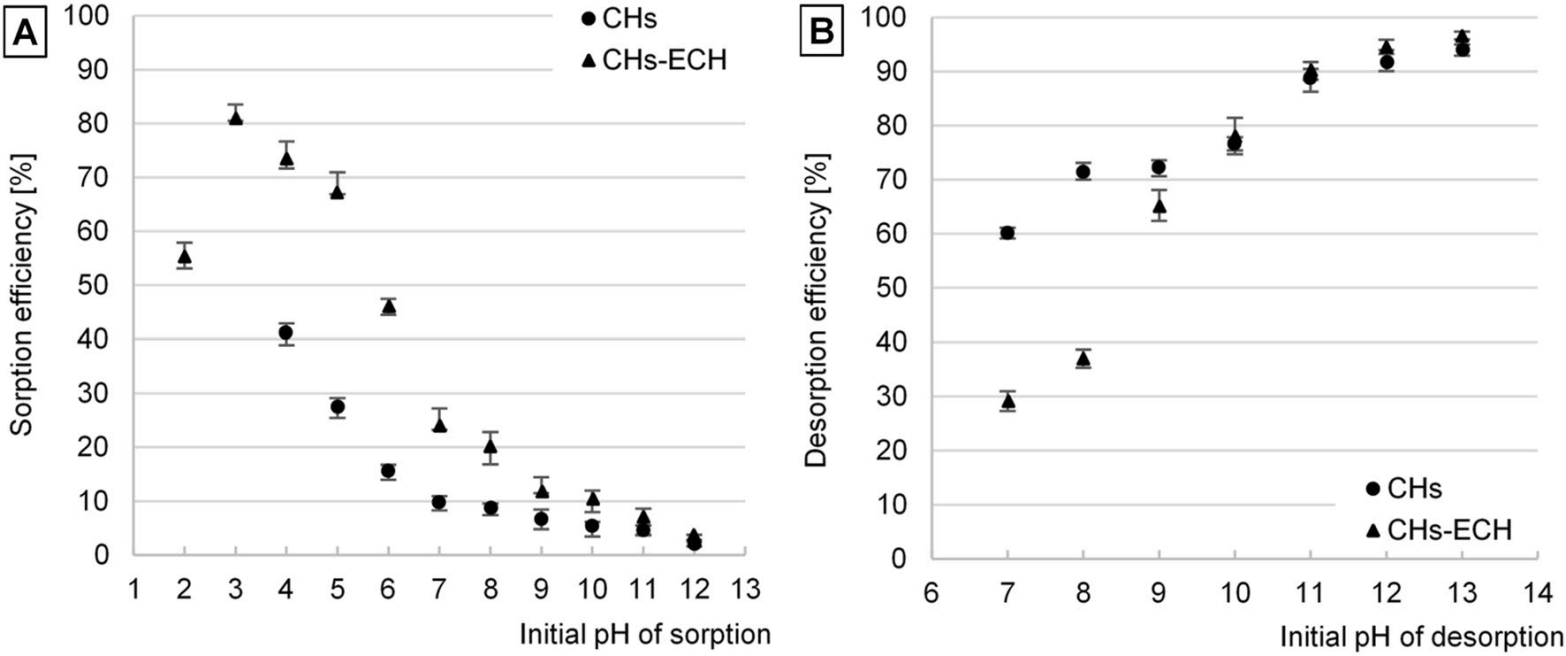
(**A**) Effect of pH on i-PO_4_ sorption efficiency, and (**B**) Effect of pH on i-PO_4_ desorption efficiency. Experiments were performed in triplicate (C_start_ = 10 mg P-PO_4_/L, sorbent dose = 1 g _d.m._/L, 150 r.p.m., temp. 22 °C).

The efficiency of orthophosphate ion (i-PO4) sorption on the sorbents tested increased along with a decreasing initial pH value of the solution (Fig. 2A). The non-crosslinked chitosan hydrogel (CHs) showed the highest sorption efficiency at pH 4. At lower pH values (pH 2–3), it swelled and dissolved, due to which it was losing its ability to bind i-PO4. In turn, the chitosan hydrogel crosslinked with epichlorohydrin (CHs-ECH) was the most effective in binding i-PO4 at pH 3. In turn, at pH 2, its sorption efficiency decreased rapidly. In the case of CHs and CHs-ECH, the lowest sorption efficiency was determined at pH 12 (Fig. 2A).

The high efficiency of orthophosphate sorption by the tested sorbents at low pH values was due to their basic nature induced by the presence of primary amine groups in chitosan’s structure. At pH < 7, the –NH_2_ groups capable of easy protonation gained a positive charge (–NH_2_ + H_3_O^+^ → –NH_3_^+^ + H_2_O). The positively-charged amine groups of the sorbents were electrostatically attracting i-PO_4_ (in the pH range of 3–7, mainly in the form of H_2_PO_4_^−^), which significantly enhanced their sorption^41^. With the initial solution’s pH increasing, the number of protonated amine groups decreased on the sorbent’s surface, which was reflected in a reduced i-PO4 binding efficiency. At pH > 10, the chitosan hydrogels gained a strong negative charge, which additionally repulsed the orthophosphate ions (in the pH range of 10–12, mainly in the form of HPO_4_^2−^) from the sorbent’s surface and hindered the sorption process^42^. The lower efficiency of orthophosphate sorption at pH 2 could be caused by the H_3_PO_4_ form of most orthophosphates at pH 2. In this form (due to the "zero" electric charge) orthophosphates react severely less with protonated amine groups of chitosan than in the ionic forms^43^. The lower efficiency of i-PO_4_ sorption on CHs-ECH at pH 2, compared to CHs-ECH sorption efficiency at pH 3, could be also due to a high number of chloride ions. The correction of orthophosphate solution’s pH to pH 2 required approximately a ten-fold higher volume of an HCl solution than its correction to pH 3. The Cl^−^ ions competed with i-PO_4_ for free sorption sites, which could impair the sorption of i-PO_4_. In turn, the higher sorption efficiency of CHs-ECH than of CHs was due to the characteristic structure of the chitosan hydrogel crosslinked with epichlorohydrin. Granules of CHs-ECH are more hydrated, have larger surface, and better permeability of the hydrogel membrane compared to CHs granules, which resulted in their higher orthophosphate ion binding efficiency of^40^.

All subsequent experiments concerning i-PO_4_ sorption on CHs were conducted at pH 4, whereas these concerning i-PO_4_ sorption on CHs-ECH—at pH 3.

The efficiency of i-PO_4_ desorption from the sorbents tested increased along with increasing pH of the desorption solution. The best desorption outcomes were achieved at pH 12–13. The mean efficiency of i-PO_4_ desorption from CHs was 94.0% and that from CHs-ECH was 96.6% (Fig. 2B).

Once sorbent had been introduced into a solution having a high pH value, the amine groups of chitosan lost their positive charge (–NH_3_^+^ + OH^−^ → –NH_2_ + H_2_O), whereas a major part of hydroxyl groups became deprotonated (–OH + OH^−^ → –O^−^ + H_2_O). Orthophosphate anions that were no longer electrostatically bound with chitosan’s amine groups were pushed out outside hydrogel’s structure due to the interaction with deprotonated hydroxyl groups.

At the initial pH 7–8, the efficiency of i-PO_4_ desorption from CHs-ECH was significantly lower compared to CHs, likely because of the various effects of the sorbents on the pH value of solutions they were in contact with and also because of various pH_PZC_ values^39^.

Both CHS and CHs-ECH changed the pH value of the sorption and desorption solutions. The system tended to reach a pH value approximating the pH_PZC_ (PZC—point of zero charge) of the sorbents tested. The pH_PZC_ determined for CHs was pH_PZC_ = 7.8 and that determined for CHs-ECH was pH_PZC_ = 6.7 (Supplement 2). Analyses concerning the effect of hydrogels on solutions’ pH and determination of sorbents’ pH_PZC_ were described in Supplement 2.

### i-PO_4_ sorption and desorption kinetics

**Figure 3:**
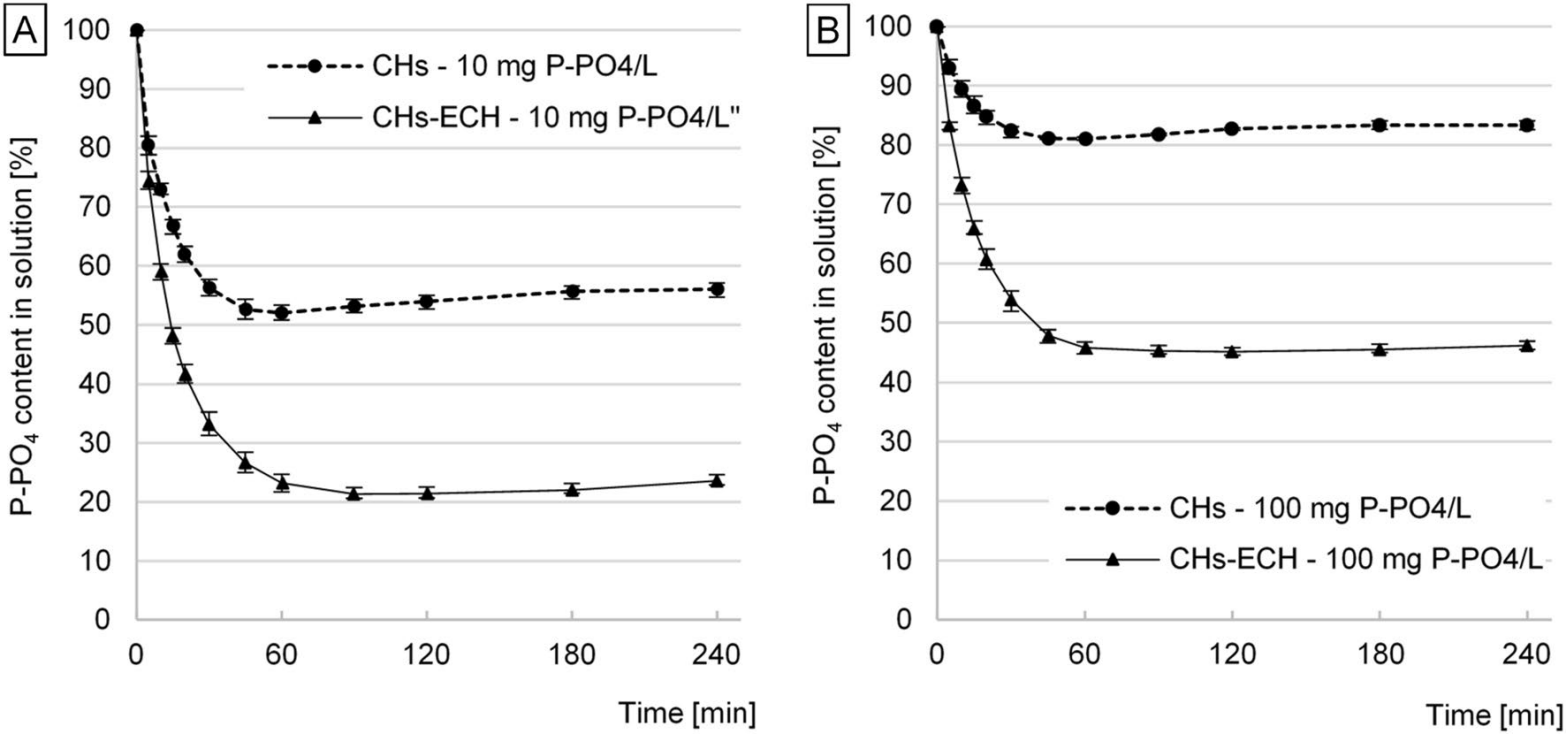
i-PO_4_ sorption kinetics. (**A**) initial concentration: 10 mg P-PO_4_/L, (**B**) initial concentration: 100 mg P-PO_4_/L. Experiments were performed in triplicate (C_start_ = 10/100 mg P-PO_4_/L, sorbent dose = 1 g _d.m._/L, sorption in pH 4 for CHs and pH 3 for CHs-ECH, 150 r.p.m., temp. 22 °C).

Regardless of the initial i-PO_4_ concentration, the time after which the amount of bound orthophosphates was the highest reached 60 min for CHs and 90 min for CHs-ECH (Fig. 3). After 60 min of sorption with CHs, the i-PO_4_ concentration in the source solution began to increase, indicating orthophosphate desorption. During sorption with CHs-ECH, the onset of i-PO_4_ release to the source solution was recorded after 120 min of the process. However, the number of lost orthophosphate ions (desorbed by CHs-ECH) was lower than in the case of CHs.

During the sorption process, the positive charge that was initially accumulated on the surface of a hydrogel granule began to be evenly distributed across the whole hydrogel’s volume. Because i-PO_4_ ions are less capable for penetrating hydrogel’ structure than protons and hydronium ions, presumably most of them were bound in the outer hydrogel layers. Because the pH value increased over the sorption process, the force of electrostatic attraction between chitosan chains and orthophosphate ions located on the sorbent’s surface decreased. Consequently, only a small part of earlier bound orthophosphate ions could be released to the source solution^16^.

In the case of CHs-ECH, the longer sorption time and the longer time after which i-PO_4_ started to be released to the source solution could be associated with the lower pH_PZC_ of the sorbent and with the lower initial sorption pH (pH3 for CHs-ECH and pH 4 for CHs). At pH 3, CHs-ECH showed higher i-PO_4_ binding ability than CHs at pH 4; however, a higher number of ions penetrating to the hydrogel granule’s interior extended the sorption process. In the experimental series with CHs-ECH, an increase in solution’s pH during i-PO_4_ sorption was not that high as in the case of CHs, which caused retardation and lesser intensity of ion release. After too long contact of the sorbent with the source solution, the phenomenon of orthophosphate desorption was also observed during nutrients removal from multi-component solutions^17^.

The kinetics of i-PO_4_ sorption on chitosan sorbents was described using pseudo-first order and pseudo-second order models (Table 1). A better fit of the pseudo-first order model to experimental data (higher values of R^2^ and smaller values of Chi square—χ^2^) is indicative of the typical physical nature of the process (physical sorption). The intensity of i-PO_4_ sorption on CHs and CHs-ECH was higher at the higher initial concentrations of the orthophosphate ions (Table 1), which can be explained by a higher probability of i-PO_4_ collisions with chitosan’s sorption centers. The higher number of orthophosphate ions additionally shifted the point of the osmotic equilibrium between i-PO_4_ concentration in the hydrogel and source solution in the system, which ultimately led to a higher absorption of the sorbate. CHs-ECH was far more effective in i-PO_4_ sorption than CHs, presumably due its specific characteristics including a larger specific surface, better permeability of the hydrogel membrane, and lower sorption pH (pH 3)^40^.

**Table 1:** Parameters of i-PO_4_ sorption and desorption kinetics determined from the pseudo-first order model and pseudo-second order model.

Sorbent	P-PO_4_ conc. (mg P-PO_4_/L)	Pseudo-first order model (non-linear)				Pseudo-second order model (non-linear)				Exp. data	
		k_1_ (1/min)	q_e,cal_ (mg/g)	R^2^	χ^2^	k_2_ (g/mg*min)	q_e,cal_ (mg/g)	R^2^	χ^2^	q_e,cal_ (mg/g)	
**Kinetic parameters of i-PO4 sorption**											
CHs	10	0.0861	4.75	0.9939	0.0423	0.0199	5.47	0.9913	0.0611	4.70	
	100	0.0855	18.81	0.9969	0.0828	0.0049	21.75	0.9853	0.3039	18.85	
CHs-ECH	10	0.0736	7.71	0.9977	0.0235	0.0093	9.13	0.9968	0.0288	7.86	
	100	0.0656	54.77	0.9986	0.1765	0.0011	55.80	0.9959	0.2591	54.75	

Sorbent	Conc. of source solut.*	Des. pH** (pH)	Pseudo-first order model (non-linear)				Pseudo-second order model (non-linear)				Exp. data
			k_1_^(desorpt.)^ (1/min)	q_e,cal_^(desorpt.)^ (mg/g)	R^2^	χ^2^	k_2_^(desorpt.)^ (g/mg*min)	q_e,cal_^(desorpt.)^ (mg/g)	R^2^	χ^2^	q_e,cal_^(desorpt.)^ (mg/g)
**Kinetic parameters of i-PO4 desorption**											
CHs	10	11	0.0985	4.19	0.9972	0.0219	0.0271	4.78	0.9950	0.0225	4.22
		12	0.2803	4.21	0.9959	0.0031	0.1337	4.44	0.9920	0.0161	4.29
		13	0.4027	4.35	0.9967	0.0119	0.2988	4.43	0.9946	0.0222	4.40
	100	11	0.0669	15.92	0.9995	0.0174	0.0039	16.12	0.9940	0.1305	15.93
		12	0.2024	15.89	0.9992	0.0122	0.0212	17.06	0.9933	0.1112	16.08
		13	0.3282	16.30	0.9943	0.0083	0.0521	16.74	0.9982	0.0133	16.56
CHs-ECH	10	11	0.0763	6.97	0.9956	0.0697	0.0147	7.61	0.9891	0.0847	7.07
		12	0.1117	7.23	0.9940	0.0795	0.0239	7.73	0.9921	0.0747	7.30
		13	0.1339	7.34	0.9977	0.0205	0.0297	7.79	0.9900	0.0741	7.42
	100	11	0.0706	44.60	0.9963	0.4795	0.0021	48.96	0.9887	0.5000	45.25
		12	0.1115	45.71	0.9966	0.2733	0.0038	48.89	0.9888	0.4607	46.50
		13	0.1195	46.82	0.9954	0.3058	0.0041	49.91	0.9884	0.4398	47.50

*Concentration of source solution (used for sorbent preparation) (mg P-PO_4_/L), **Desorption pH. (C_start_ = 10/100 mg P-PO_4_/L, sorbent dose = 1 g _d.m._/L, sorption in pH 4 for CHs and pH 3 for CHs-ECH, desorption at pH 11–13, 150 r.p.m., temp. 22 °C.).

**Figure 4:**
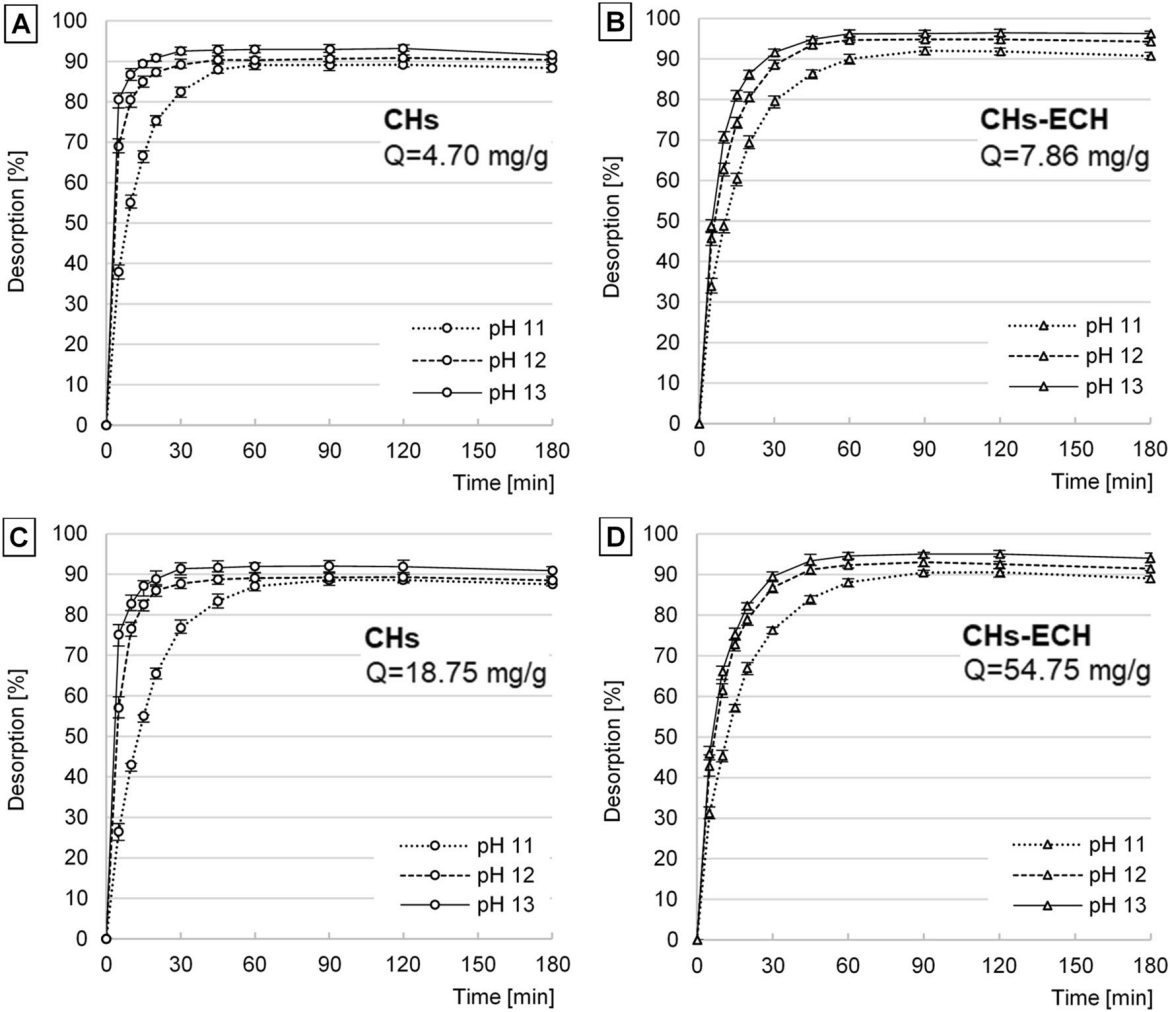
i-PO4 desorption kinetics at pH 11–13. (**A**) CHs—sorbent prepared at the source solution concentration of 10 mg P-PO_4_/L (Q = 4.70 mg/g). (**B**) CHs-ECH—sorbent prepared at the source solution concentration of 10 mg P-PO_4_/L (Q = 7.86 mg/g). (**C**) CHs—sorbent prepared at the source solution concentration of 100 mg P-PO_4_/L (Q = 18.85 mg/g). (**D**) CHs-ECH—sorbent prepared at the source solution concentration of 100 mg P-PO_4_/L (Q = 54.75 mg/g). Experiments were performed in triplicate. (Sorbent dose = 1 g _d.m._/L, desorption at pH 11–13, 150 r.p.m., temp. 22 °C).

The time needed to reach the highest i-PO_4_ desorption depended on sorbent type, the concentration of the source solution used to prepare the sorbent, and pH of the desorption solution. In the experimental series in which CHs was prepared in the source solution with C_start_ = 10 mg P-PO_4_/L, the maximum i-PO_4_ concentration in the desorption solution was achieved after 30 min at desorption process pH 11 and after 45 min at pH 12–13 (Fig. 4A). In turn, in the experimental series in which CHs was prepared in the source solution with C_start_ = 100 mg P-PO_4_/L, desorption times were longer and reached 60 min at desorption pH 11 and 45 min at desorption pH 12 and pH 13 (Fig. 4C). In the case of CHs-ECH prepared in the solution with C_start_ = 10 mg P-PO_4_/L, desorption times were from 60 min at pH 12 and pH 13 to 90 min at pH 11. With CHs-ECH prepared in the source solution with C_start_ = 100 mg P-PO_4_/L, desorption times noted at pH 12 and 13 elongated to 90 min (Fig. 4B, D).

At higher pH values of the desorption process, the sorbents had a higher total negative charge. The stronger electrostatic repulsion between chitosan chains and orthophosphate anions significantly shortened desorption time.

Differences between the duration of orthophosphate desorption from the sorbents prepared in source solutions with different i-PO4 concentrations (10 and 100 mg P-PO_4_/L) are probably due to the various amounts of orthophosphate ions bound. At the source solution concentration of C_start_ = 10 mg P-PO_4_/L, Q_max_ was 4.70 mg/g (for CHs) and 7.86 mg/g (for CHs-ECH), whereas at the concentration of C_start_ = 100 mg P-PO_4_/L, Q_max_ reached 18.85 mg/g (for CHs) and 54.75 mg/g (for CHs-ECH) (Table 1). The higher amount of bound i-PO4 ions caused an extended time of their desorption.

A similar explanation can be provided for differences between i-PO4 desorption times from CHs and CHs-ECH. Before desorption, the crosslinked sorbent possessed a significantly higher number of sorbed orthophosphate ions than CHs, which could prolong the desorption process. Besides, the longer i-PO4 desorption from CHs-ECH could be affected by the characteristic swollen structure of the crosslinked hydrogel^40^ and lower pH used during sorbent preparation. Due to the greater diameter of CHs-ECH granules saturated with orthophosphate ions and also because of chloride ions (pH correction with HCl), the time needed for a change in the charge of the chitosan chains in deeper sorbent layers was considerably longer. This could, additionally, retard desorption of i-PO4 located inside the sorbent.

In most of the experimental series, a small decrease was noted in i-PO4 concentration in the desorption solution after 120 min, which could be associated with a partial decrease in desorption solution pH and a change in the charge on sorbent’s surface. The lower total negative charge on sorbent’s surface probably influenced the shift of osmotic equilibrium of the system. As a result, a small part of earlier desorbed orthophosphate ions was again bound with the chitosan hydrogel.

Like sorption kinetics, the kinetics of desorption process was described with the pseudo-first order and pseudo-second order models (Table 1). The best fit of experimental data to the pseudo-first order model (higher values of R^2^ and smaller values of Chi square—χ^2^) confirms that desorption process’s mechanism was based on physical interactions.

The intensity of i-PO4 desorption from the sorbents tested increased along with pH increase (Fig. 4, k1 values—Table 1). Even though the pH value of desorption solutions had a significant effect on i-PO4 desorption rate, the effect of pH (pH 11–13) on the ultimate amount of i-PO4 desorbed was relatively small (Table 1).

In the experimental series with the same pH value of the desorption solution, the percentage efficiency of i-PO4 desorption from CHs and CHs-ECH was similar (Fig. 4). However, CHs-ECH’s capability for binding more i-PO4 from the source solutions compared to CHs resulted in ultimately higher concentrations of the orthophosphates in desorption solutions (Table 1).

The sorption/desorption time established in the successive experimental series reached 60 min/60 min for CHs and 90 min/90 min for CHs-ECH.

### Effect of sorbent time and source solution concentration on the efficiency of cyclic i-PO4 sorption/desorption

**Figure 5:**
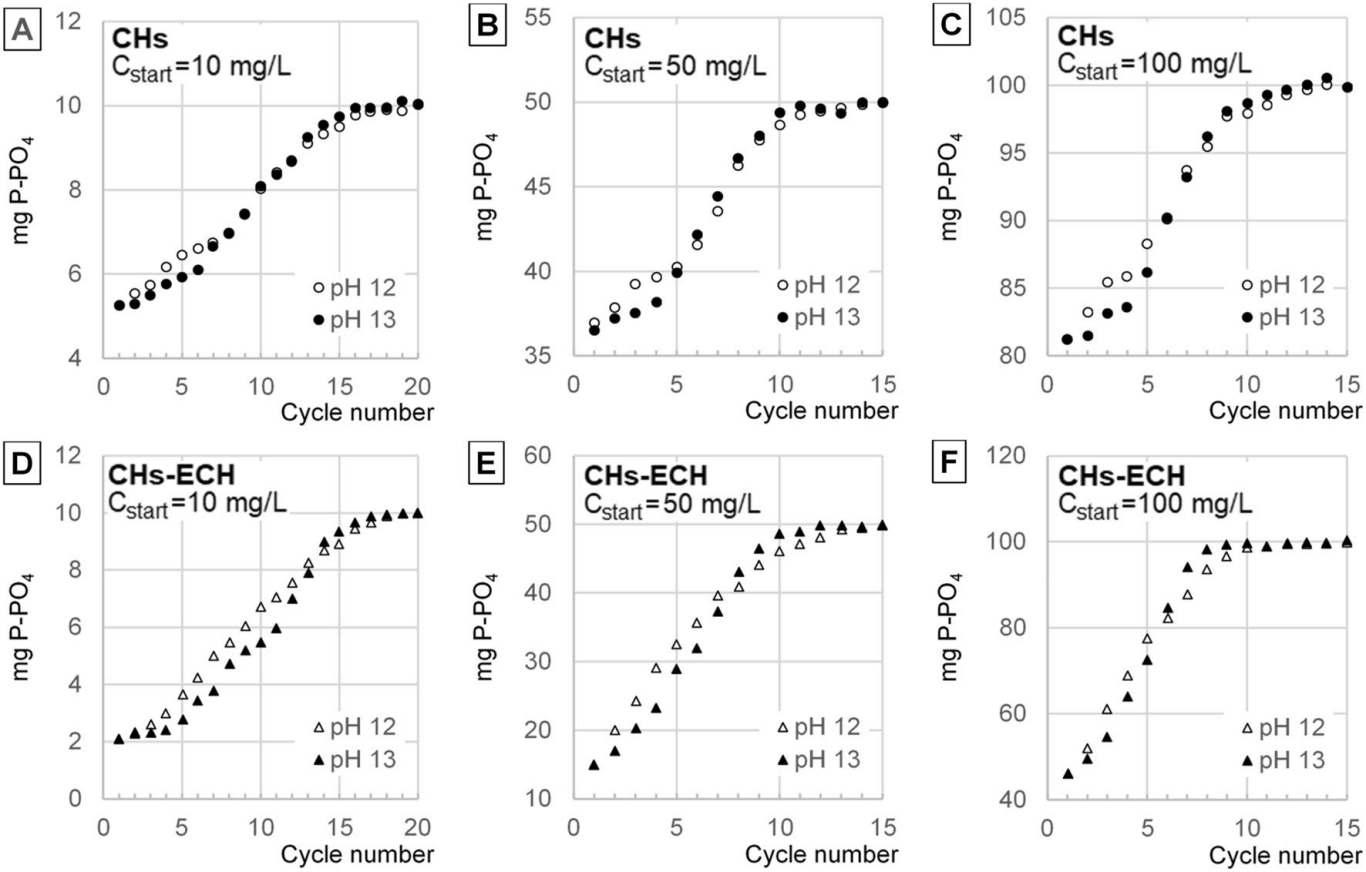
Concentrations of i-PO4 in source solutions (C_start_ = 10–100 mg P-PO_4_/L) after sorption in the successive cycles using CHs and CHs-ECH : (**A**) CHs, C_start_ = 10 mg P-PO_4_/L, (**B**) CHs, C_start_ = 50 mg P-PO_4_/L, (**C**) CHs, C_start_ = 100 mg P-PO_4_/L, (**D**) CHs-ECH, C_start_ = 10 mg P-PO_4_/L, (**E**) CHs-ECH, C_start_ = 50 mg P-PO_4_/L, (**F**) CHs-ECH, C_start_ = 100 mg P-PO_4_/L. (Sorbent dose = 1 g _d.m._/L, sorption in pH 4 for CHs and pH 3 for CHs-ECH, desorption at pH 12 and 13, 150 r.p.m., temp. 22 °C).

**Figure 6:**
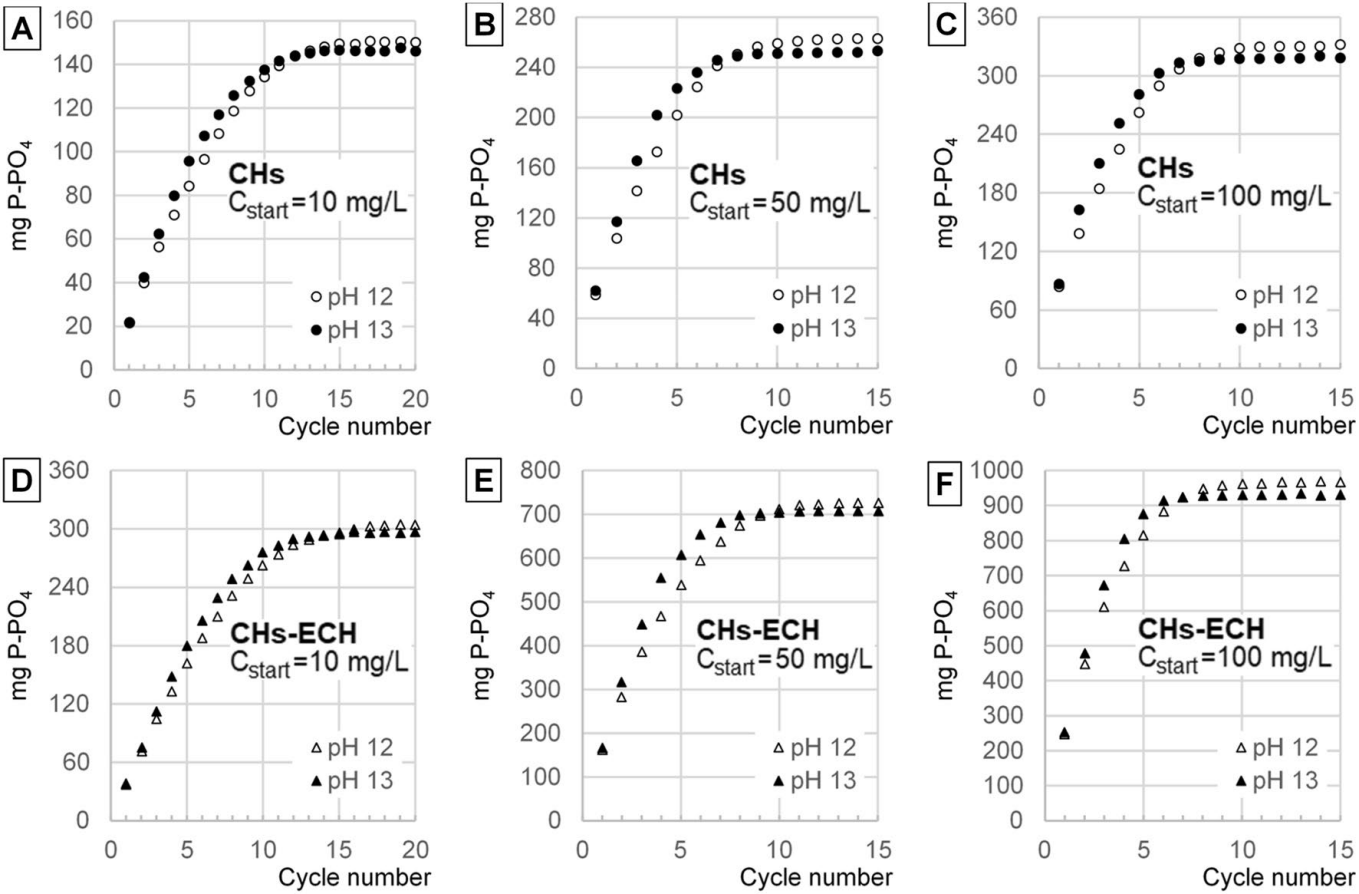
Concentrations of i-PO4 in the concentrate after desorption in the successive cycles: (**A**) CHs, C_start_ = 10 mg P-PO_4_/L, (**B**) CHs, C_start_ = 50 mg P-PO_4_/L, (**C**) CHs, C_start_ = 100 mg P-PO_4_/L, (**D**) CHs-ECH, C_start_ = 10 mg P-PO_4_/L, (**E**) CHs-ECH, C_start_ = 50 mg P-PO_4_/L, (**F**) CHs-ECH, C_start_ = 100 mg P-PO_4_/L. (Sorbent dose = 1 g _d.m._/L, sorption in pH 4 for CHs and pH 3 for CHs-ECH, desorption at pH 12 and 13, Source solution volume to concentrate volume ratio—5:1, 150 r.p.m., temp. 22 °C).

The concentration of orthophosphate ions increased in source solutions with each sorption cycle, indicting decreasing i-PO4 sorption efficiency on the sorbents decreased in the successive cycles (sorption/desorption). The number of cycles in which the sorbents could bind i-PO4 decreased along with an increasing concentration of the source solution. The sorbents tested were losing their sorptive abilities faster in the systems with higher desorption pH (Figs. 5, 6).

In the first cycles, i-PO4 sorption from the source solution was more efficient in the systems where desorption proceeded at higher pH (pH 13). In the last cycles, however, the efficiency of orthophosphate binding from the source solution was higher in the series with lower desorption pH (pH 12) (Figs. 5, 6).

The concentration of i-PO4 in the orthophosphate concentrate increased with each sorption/desorption cycle, but desorption efficiency decreased. The concentrate’s concentration increased until the sorbent batch lost its sorptive ability.

The pH value of the desorption process had a moderate effect on the concentration of orthophosphates in the concentrate (Fig. 6). In the initial cycles, the i-PO4 concentration was higher in the concentrates with pH 13, indicating a higher desorption efficiency than in the systems with pH 12. However, in the successive cycles, the efficiency of i-PO4 desorption in the systems with the concentrate having pH 13 decreased significantly faster than in these with the concentrate having pH 12. Ultimately, in the experimental series with desorption conducted at pH 12, the maximum concentration of orthophosphates was higher than in the systems with pH 13. The differences in the maximum concentrations between orthophosphate concentrates with pH 12 and pH 13 were, however, small (Fig. 6).

The concentrations of the concentrates increased along with an increasing concentration of the source solutions of i-PO4. The concentrations of i-PO4 in the concentrate were also higher in the experimental series with CHs-ECH used as the carrier of these ions. The maximum concentration of orthophosphates obtained in the concentrate ranged from 150.0 mg/L (CHs) and 304.2 mg/L (CHs-ECH) at a source solution concentration of C_start_ = 10 mg P-PO_4_/L, to 332.0 mg/L (CHs) and 971.6 mg/L (CHs-ECH) at a source solution concentration of C_start_ = 100 mg P-PO_4_/L (Fig. 6; Table 2).

**Table 2:** Parameters of orthophosphate concentrate in the last sorption/desorption cycle.

Sorbent	Conc. of P-PO4 in source solution (mg/L)	pH during desorption	Conc. Of P-PO4 in concentrate (mg/L)	Chloride conc. (mg Cl^−^/L)	TDS in concentrate (mg Cl^−^/L)	pH of concentrate	Dry residue* (mg/L)	P-PO4 content in the sediment** (%)
CHs	10	pH 12	150.01	16.5	805	11.98	842.19	17.81
		pH 13	146.02	20.9	1111	12.99	1196.2	12.21
	50	pH 12	262.84	11.3	1259	11.76	1390.86	18.90
		pH 13	252.35	15.0	1489	12.99	1625.11	15.53
	100	pH 12	331.98	9.47	1556	11.67	1674.11	19.83
		pH 13	318.31	16.4	1788	12.92	1889.26	16.85
CHs-ECH	10	pH 12	304.24	326	1801	9.38	1896.88	16.04
		pH 13	296.96	377	2599	12.9	2670.5	11.12
	50	pH 12	723.84	191	4106	8.56	4187.52	17.29
		pH 13	706.39	249	4682	12.69	4819.46	14.66
	100	pH 12	971.61	106	5321	8.42	5477.32	17.74
		pH 13	931.06	149	5896	12.59	6049.74	15.39

*Dry residue (after concentrate evaporation). **P–PO_4_ content in the sediment after evaporation of the concentrate (105 °C).

The efficiency of i-PO4 desorption to the concentrate decreased with each cycle due to increasing concentrations of orthophosphates and chlorides in the concentrate. Desorption proceeded in the successive cycles until osmotic equilibrium had been reached between i-PO4 concentration in the hydrogel and in the concentrate^21^. With each successive cycle, an increasing number of non-desorbed anions (i-PO4, Cl^−^) was left in the sorbent after desorption. Thus, the number of free sorption centers able to bind orthophosphates from the source solution decreased continuously in the successive cycles, leading to sorption efficiency decrease with each cycle (Fig. 5). No increase in i-PO4 concentration in the hydrogel in the last cycles caused no increase in their concentration in the concentrate (Fig. 6).

Concentrations of the concentrates obtained increased along with an increasing concentration of the source solution because the sorption efficiency increasing along with concentration increase. At the higher concentration of orthophosphate ions in the hydrogel, the osmotic equilibrium point allowed for a higher i-PO4 concentration in the concentrate. For the same reason, a higher concentration of the concentrate was obtained in the systems with CHs-ECH (Fig. 6).

The higher concentrations of i-PO4 determined in the concentrates in the first cycles at higher desorption pH (pH 13) are due to a stronger negative charge of the sorbent, which enhanced desorption of orthophosphates. For the same reason, in the initial cycles of the experimental series with desorption performed at pH 13, the efficiency of i-PO4 sorption from the source solutions was higher than in the series with desorption conducted at pH 12. Concentrate’s pH was corrected (to pH 12/pH 13) after each sorption/desorption cycle. Therefore, salinity and TDS content determined in the final cycles were significantly higher in the concentrates with pH 13 than in these with pH 12, which could negatively affect orthophosphate desorption. Hence, in the last cycles, the maximum concentration of orthophosphates obtained in the concentrates with pH 12 was ultimately higher than in these with pH 13.

Supplement 3 presents analyses on the effect of sorbent type and source solution concentration on TDS contents in the concentrates and source solutions.

Supplement 4 presents analyses on the effect of sorbent type on the pH values of source solutions and concentrates in the successive sorption/desorption cycles.

### Parameters of the orthophosphate concentrate in the last sorption/desorption cycle

In the last sorption/desorption cycle, the concentrate was analyzed for the concentration of chlorides. The concentrates obtained in the systems with CHs used as the ion carrier were characterized by multiply higher Cl^−^ concentrations than these with CHs-ECH (Table 2). Chloride concentrations in the orthophosphate concentrates decreased along with an increase in the initial concentration of the source solutions. Besides, they were higher in the experimental series with desorption performed at the higher pH value (pH 13) (Table 2).

The higher chloride concentrations in the series with CHs-ECH were due to the lower pH of the sorption process. Because pH value was corrected using HCl, the source solutions from the systems with CHs-ECH (pH 3) had approximately tenfold higher chloride concentrations than these from the systems with CHs (pH 4). At the higher concentrations of chlorides, their sorption efficiency was higher, which resulted in their higher concentration in the concentrates.

The decreased concentration of Cl^−^ in the concentrates along with an increasing initial concentration of i-PO4 in the source solutions was due to the lower chloride to orthophosphate ratio in the solution. At the higher concentrations of orthophosphates during the sorption process, the ratio of bound orthophosphates to bound chlorides increased, which caused a lower number of Cl^−^ anions in the concentrate. The higher concentrations of chlorides in the experimental series with desorption conducted at pH 13 are due to a stronger negative charge of the sorbent in the concentrate and more efficient desorption. Important was also the lesser affinity of chlorides to the amine groups of chitosan^22^, which additionally increased their desorption efficiency.

The mass of the recovered phosphorus, computed based on concentrate’s concentration, increased along with an increasing concentration of the source solution (Table 2). The effect of desorption pH on phosphorus content in the concentrate was insignificant. The amounts of P-PO_4_ recovered in the systems with CHs-ECH were significantly higher compared to the systems with CHs. Phosphorus content determined in the concentrate in the experimental series with CHs-ECH used as the ion carrier was even 30-fold higher than in the source solutions (at C_start_ = 10 mg P-PO_4_/L).

The content of dry residue left after concentrate evaporation depended mainly on TDS content in the solution. It increased along with an increasing concentration of the source solution. The content of concentrate dry residue obtained in the last cycle was significantly higher in the experimental series with CHs-ECH than in these with CHs (Table 2). Dry residue contents were also higher in the systems with higher desorption pH (pH 13).

The percentage contents of phosphorus (P-PO_4_) in the dry residue after concentrate evaporation increased along with an increasing initial concentration of the source solutions. They were strongly affected by desorption pH. In the experimental series with the lower desorption pH (pH 12), the purity of the recovered phosphorus was significantly higher than in these with the higher desorption pH (pH 13).

The percentage contents of phosphorus (P-PO_4_) in the dry residue after concentrate evaporation were generally higher in the systems with CHs than with CHs-ECH (Table 2). At desorption pH 12, they ranged from 17.81 to 19.83% in the systems with CHs and from 16.04 to 17.74% in the systems with CHs-ECH.

The percentage contents of phosphorus (P-PO_4_) in the dry residue after concentrate evaporation were mainly due to the ratio of i-PO4 to TDS contents in the concentrate. In turn, the effect of sorption and desorption parameters on i-PO4 and TDS contents in the concentrate was explained in the previous paragraphs of this section (and also in Supplement 3).

After physicochemical analyses, the solubility of the sediment after concentrate evaporation was determined by mixing it with a small volume of deionized water. In contact with water, the sediment dissolved completely, which points to the utile form of the recovered phosphorus.

## Summary

The content of phosphorus in the dry residue left after concentrate evaporation reached max. 17.74% in the experimental series with CHs-ECH and even 19.83% in these with CHs, suggesting that the recovered phosphorus was mainly in the form of sodium phosphates and hydrophosphates. The total solubility of the concentrate dry residue in a small volume of water points to the utile and convenient form of the recovered phosphorus.

The maximum concentration of orthophosphates in the concentrate ranged from 146.0 to 971.6 mg P-PO_4_/L and depended mainly on the initial i-PO_4_ concentration in the source solutions and ion carrier type (sorbent type). Due to the better sorption efficiency of orthophosphates on CHs-ECH than on CHs, the experimental series with crosslinked chitosan were also characterized by higher concentrations of the concentrate obtained.

The sorption of orthophosphates on the crosslinked chitosan sorbents was the most efficient at pH 3. Considering the dissolution of non-modified chitosan at pH < 4, the optimal pH of i-PO_4_ sorption was pH 4.

The sorption of orthophosphates on CHs-ECH at low pH (pH 3) resulted in very high TDS contents in the concentrates. Because the concentration of chlorides was approximately tenfold higher in the source solutions with pH 3 (pH correction using HCl) than in these with pH 4, the Cl^−^ sorption on CHs-ECH was significantly higher than on CHs. As a consequence, CHs-ECH introduced greater amounts of chlorides to the concentrate than CHs. Also, the higher concentration of chlorides in the concentrate required higher amounts of NaOH to correct concentrate’s pH after each cycle. The higher Cl^−^ and Na^+^ concentrations in the concentrate increased its salinity and TDS content. Ultimately, the concentrates obtained in the experimental series with CHs-ECH were more contaminated with Cl^−^ and Na^+^ ions.

The efficiency of orthophosphate desorption increased along with increasing pH of desorption solution. In the case of both CHs and CHs-ECH, desorption efficiency was the highest at pH 12–13. Even though the desorption process was more intense at high pH of desorption solutions, the final amounts of orthophosphates desorbed at pH 11, pH 12, and pH 13 were similar, pointing to no need for the use of strongly alkaline solutions to perform effective desorption.

In the initial sorption/desorption cycles, the concentration of orthophosphates in the concentrate at pH 13 was higher than in the series with the concentrate at pH 12. Due to the rapid salination of the concentrate at pH 13 in the successive cycles, ultimately higher maximum concentrations of the concentrate were obtained in the experimental series with desorption performed at pH 12. The lower concentration of orthophosphates in the concentrates at pH 13 and their higher salt content suggest that P-PO_4_/L desorption should be conducted at relatively low pH values (pH < 13).

The process of orthophosphate binding on chitosan sorbents was also largely influenced by the sorbent’s contact time with the solution. In case of CHs, it should range from 45 to 60 min, whereas in the case of CHs-ECH from 90 to 180 min. Longer contact times could result in uncontrolled desorption of i-PO_4_ to the source solution.

The optimal duration of i-PO_4_ desorption from chitosan sorbents depended mainly on desorption pH. It shortened along with desorption pH increase, due to a stronger negative charge of the sorbent at higher desorption pH. Regardless of the concentration of source solutions, i-PO_4_ desorption from CHs should last from 60 to 120 min and that from CHs-ECH—from 90 to 120 min. A too long contact time of the sorbent with the desorption solution could result in re-resorption of orthophosphates, that had earlier been released to the solution.

## Supplementary Information

Supplementary Information.
