# Supplementary material for: Recovery of phosphorus as soluble phosphates from aqueous solutions using chitosan hydrogel sorbents
Source: Sci Rep. 2021 Aug 18;11:16766. doi: 10.1038/s41598-021-96416-2 (PMC8373865; doi:10.1038/s41598-021-96416-2)
Supplement: Supplementary file 1 — Supplementary Information. [file 41598_2021_96416_MOESM1_ESM.docx]

**Recovery of phosphorus as soluble phosphates from aqueous solutions using chitosan hydrogel sorbents**

Tomasz Jóźwiak^1a*^, Agata Kowalkowska^1b^, Urszula Filipkowska^1c^, Joanna Struk-Sokołowska^2d^, Ludmila Bolozan^3e^, Luminita Gache^3f^, Marius Ilie^3g^

^1^ Department of Environmental Engineering, University of Warmia and Mazury in Olsztyn, Warszawska St. 117a, 10-957 Olsztyn, Poland

^2^ Department of Environmental Engineering Technology and Systems, Bialystok University of Technology, Wiejska St. 45E, Bialystok 15-351, Poland

^3^ Faculty of Chemical Engineering and Environmental Protection, Gheorghe Asachi Technical University of Iaşi, Bulevardul Profesor Dimitrie Mangeron 67, Iași 700050, Romania

^a^ tomasz.jozwiak@uwm.edu.pl, ^b^ agata.kowalkowska19@wp.pl,
^c^ urszula.filipkowska@uwm.edu.pl,  ^d^ j.struk@pb.edu.pl, ^e^ liuda.bolozan1234@gmail.com,
^f^ gacheluminita1995@gmail.com, ^g^ ilie_marius26@yahoo.com

* Corresponding author: Tomasz Jóźwiak, Department of Environmental Engineering, University of Warmia and Mazury in Olsztyn, ul. Warszawska 117a, 10-957 Olsztyn, Poland, e-mail: tomasz.jozwiak@uwm.edu.pl

**Supplement 1**

**IR spectra of the analyzed chitosan sorbents**


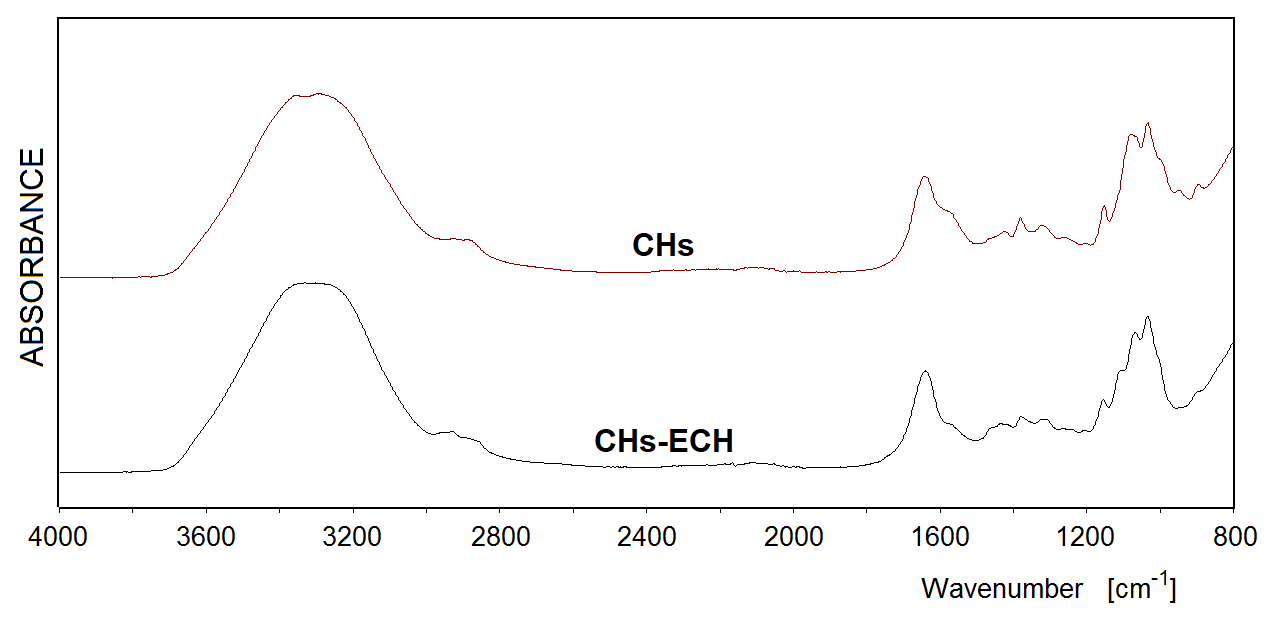


Fig. S1. IR spectra of CHs and CHs-ECH. (made using the ALPHA FT-IR-base spectrometer – by BRUKER (Germany))

CHs and CHs-ECH have saccharide structure, what is indicated by peaks at 950 cm^−1^, 1075 cm^−1^ and 1320 cm^−1^ (stretching C-O bonds), as well as at 1020 cm^−1^ (stretching C-O-C bonds) ^1^. Peaks at 1152 cm^-1^ and 1640 cm^-1^ (vibration of N-H bond) suggest the presence of the amino group in hydrogel structure. The wide band at 3000-3600 cm^-1^ (vibrationof O-H bond) is an indicator of the hydroxyl group ^2^. No clear peak at 1560 cm^-1^ on the CHs-ECH spectrum indicates that epichlorohydrin does not react with amino groups during crosslinking ^3^. Characteristic peak at 1106 cm^-1^ (a primary alcohol group) suggests, that hydroxyl chitosan groups take part in the crosslinking reaction  ^4^.

1. Mitra, T., Sailakshmi, G., Gnanamani, A. & Mandal, A. B. Studies on cross-linking of succinic acid with chitosan/collagen. *Mater. Res.* **16**, 755–765 (2013).

2. Sowmya, A. & Meenakshi, S. A novel quaternized chitosan-melamine-glutaraldehyde resin for the removal of nitrate and phosphate anions. *Int. J. Biol. Macromol.* **64**, 224–232 (2014).

3. Coelho, T. C., Laus, R., Mangrich, A. S., de Fávere, V. T. & Laranjeira, M. C. M. Effect of heparin coating on epichlorohydrin cross-linked chitosan microspheres on the adsorption of copper (II) ions. *React. Funct. Polym.* **67**, 468–475 (2007).

4. Wei, Y. C., Hudson, S. M., Mayer, J. M. & Kaplan, D. L. The crosslinking of chitosan fibers. *J. Polym. Sci. Part A Polym. Chem.* **30**, 2187–2193 (1992).

**Supplement 2**

**Analyses of sorbent effect on solutions’ pH value after sorption and desorption**

Fig. S2. A) Effect of sorbents on changes in solutions’ pH after sorption. B) Effect of sorbents on changes in solutions’ pH after desorption, C) Determination of pH_PZC_ of the sorbents (titration method). (C_start_=10 mg P-PO_4_/L, sorbent dose = 1 g _d.m._/L, 150 r.p.m., temp. 22 ^o^C.).

Both CHs and CHs-ECH caused changes in pH values of sorption and desorption solutions (Fig. S2). The system tended to reach the pH value approximating the point of zero charge (pH_PZC_) of the sorbents tested. The pH_PZC_ determined for CHs was pH_PZC_=7.8 and that for CHs-ECH was pH_PZC_=6.7 (Fig. S2. C).

At the low initial pH (pH < pH_PZC_), amine ions present in chitosan’s structure seized the proton present in the hydronium cation, leading to the protonation of amine groups and to a decrease in hydronium ion concentration in the solution (-NH_2_ + H_3_O^+^ 🡪 -NH_3_^+^ + H_2_O). The reduction in H_3_O^+^ (H^+^) concentration resulted in solution’s pH increase (Fig. S2. C). The increase in pH values during sorption could also be due to the increasing Na^+^ to H_2_PO_4_^-^/HPO_4_^2-^ ion ratio in the solution. At the higher initial pHs (pH > pH_PZC_), hydroxyl anions present in the solution seized the proton of the hydroxyl group (-OH + OH^-^ 🡪 -O^-^ + H_2_O), which led to the deprotonation of **hydroxyl groups** (-O^-^) and to the conversion of **hydroxyl ions** into water molecules. The lower concentration of OH- ions decreased solution’s pH (Fig. S2. C). A decrease in desorption solution’s pH could also be due to an increasing concentration of i-PO4 ions which, additionally, acidified the solution.

The lower pH_PZC_ value determined for CHs-ECH (pH_PZC_=6.7), than for CHs (pH_PZC_=7.80), could be affected by the presence of hydrochloric acid in crosslinked hydrogel granules, which was a by-product of chitosan crosslinking with epichlorohydrin.

The CHs-ECH exhibited a significantly higher potency to reduce pH of the desorption solution than CHs (Fig. S2. B), mainly due to the lower sorption pH used during sorbent preparation for analyses and to a higher amount of orthophosphate ions sorbed. Once the sorbents were prepared for experiments at pH 3, the CHs-ECH bound a significantly higher amount of orthophosphate and chloride ions than CHs at pH 4. Compared to CHs, the CHs-ECH released more i-PO4 and Cl^-^ ions during desorption, which reduced desorption solution’s pH. This reduction could also be affected by hydrochloric acid present in CHs-ECH hydrogel granules as a residue of the crosslinking process.

**Supplement 3**

**Analyses of the effect of sorbent type and source solution’s concentration on TDS content in the concentrates and source solutions**

The total dissolved solids (TDS) included mainly orthophosphate and chloride anions, and sodium cations.

The TDS content in the source solutions was mainly due to the presence of sodium hydrophosphate and hydrochloric acid (pH correction). In the case of concentrates, apart from the ions carried onto sorbents from source solutions, the TDS content was increased by the addition of sodium hydroxide (pH correction).

The TDS content increased in the source solutions after sorption in the successive cycles (Fig. S3. A-F). It was also observed to increase along with an increasing initial concentration of the source solutions. Generally, higher TDS contents were determined in the source solutions after sorption in the systems with desorption performed at pH 13 and these with CHs-ECH used as carriers of ions (Fig. S3. A-F).

The TDS content in the concentrate increased with each cycle, with the highest increase noted in the first 5-7 cycles (Fig. S3. G-L), and also with an increasing concentration of the source solution.

The TDS content in the concentrate was always higher in the experimental series in which the concentrate’s pH was corrected to pH 13. Higher TDS contents were also determined in the systems with CHs-ECH used as the ion carrier.

The maximal TDS content obtained in the concentrate after the last cycle ranged from 1111 ppm (CHs, pH 13) and 1801 ppm (CHs-ECH, pH 13) at the source solution concentration of C_start_=10 mg P-PO_4_/L to 1788 ppm (CHs, pH 13) and 2599 ppm (CHs-ECH, pH 13) at the source solution concentration of C_start_=10 mg P-PO4/L (Fig. S3. G-L).

Fig. S3. **[A-F] - TDS content in the source solutions (C_start_=10-100 mg P-PO_4_/L) after sorption in the successive cycles using CHs and CHs-ECH** : A) CHs, C_start_=10 mg P-PO_4_/L, B) CHs, C_start_=50 mg P-PO_4_/L, C) CHs, C_start_=100 mg P-PO_4_/L, D) CHs-ECH, C_start_=10 mg P-PO_4_/L, E) CHs-ECH, C_start_=50 mg P-PO_4_/L, and F) CHs-ECH, C_start_=100 mg P-PO_4_/L. **[G-I] – TDS content in the concentrate after desorption in the successive cycles:** G) CHs, C_start_=10 mg P-PO_4_/L, H) CHs, C_start_=50 mg P-PO_4_/L, I) CHs, C_start_=100 mg P-PO_4_/L, J) CHs-ECH, C_start_=10 mg P-PO_4_/L, K) CHs-ECH, C_start_=50 mg P-PO_4_/L, and L) CHs-ECH, C_start_=100 mg P-PO_4_/L. (Sorbent dose = 1 g _d.m._/L, sorption in pH 4 for CHs and pH 3 for CHs-ECH, desorption at pH 12 and 13, Source solution volume to concentrate volume ratio – 5:1, 150 r.p.m., temp. 22 ^o^C).

The increasing TDS content in the concentrates in the successive cycles was due to the increasing concentrations of orthophosphates and chlorides and to pH correction with NaOH (before each desorption). The TDS content increase in the concentrates in the last cycles was small because of the low desorption efficiency. New ions appearing in the concentrate were mainly derived from NaOH delivered to the system. Its higher dose used for pH correction resulted in a higher TDS content in the concentrate, which explains higher TDS content in the concentrate with pH 13 than in that with pH 12 (Fig. S3. G-L).

The higher TDS contents in the concentrates in the systems with CHs-ECH than in these with CHs were due to, i.a., a higher capability of the crosslinked sorbent for ion transport. In addition, the higher TDS contents noted in the experimental series with CHs-ECH were also associated with lower sorption pH in the source solution and a higher capability of the crosslinked hydrogel for modifying concentrate’s pH. The source solution’s pH correction to pH 3 (sorption with CHs-ECH) required approximately ten times more HCl than pH correction to pH 4 (sorption with CHs), which caused a higher initial TDS content in the source solutions (Fig. S3. A-F). In the systems with CHs-ECH, the sorbent bound higher amounts of not only orthophosphates but also chlorides which were ultimately introduced into the concentrate. Besides, in the systems with CHs-ECH, the concentrates required significantly more NaOH during pH correction than the systems with CHs, which additionally increased TDS content in the desorption solution.

The sorbents’ sorptive capability decreasing in the successive cycles resulted in a higher number of ions left in the source solutions, which explains the increasing TDS content in source solutions in the subsequent cycles (Fig. S3. A-F). This increase could also be due to a higher amount of sodium cations carried onto sorbents from the concentrate. During desorption, the sorbents not only released orthophosphate ions to the concentrate but also bound a small amount of sodium cations from the solution. The Na^+^ ions could be released to the source solutions due to the subsequent change of the sorbent’s surface charge. Because sodium concentration increased in the concentrate in the successive cycles, an increase was also observed in the amount of sodium cations transported to the source solutions. The efficiency of sodium ion release to the source solutions depended on the content of cations in the solutions and was the highest in the case of the lowest initial concentration. Ultimately, at the source solution concentration of C_start_=10 mg P-PO_4_/L, the TDS content after sorption was higher in the last cycles compared to its initial value (Fig. S3. A,D). Because of the less intense release of sodium ions at the higher cation concentrations in the solution, the above effect was not achieved in the experimental series with the source solution concentration of C_start_=50/100 mg P-PO_4_/L.

**Supplement 4**

**Analyses of the effect of CHs and CHs-ECH on the pH values of source solutions and concentrates in the successive sorption/desorption cycles**

The pH value of the source solutions increased after sorption in the successive cycles (Fig. S4. A-F). In general, higher pH values of the solution were obtained after sorption in the experimental series with the higher desorption pH (pH 13). The higher pH values of the source solutions were determined after sorption also in the systems with CHs-ECH used as the ion carrier (Fig. S4. D-F). The pH value of the source solutions increased also along with an increasing initial concentration of orthophosphates, which was especially tangible in the experimental series with CHs-ECH (Fig. S4. D-F).

As mentioned in the Methods section, the pH value of the concentrate was corrected to the initial value (pH 12 or pH 13 depending on the experimental series) after each sorption/desorption cycle. The pH changes triggered by sorbent’s contact with the concentrate were the greatest in the initial cycles. The difference between the initial and final pH value of the concentrate was diminishing in the successive cycles, which resulted in successively decreasing NaOH concentration necessary for pH correction (to the initial value).

The changes in concentrate’s pH were the smallest in the cycles in which the hydrogel sorbents were no longer capable of binding orthophosphates. The extent of pH changes in the concentrates increased in the successive cycles along with the initial concentration of the source solution. The pH value of the desorption solution after sorption was also largely influenced by the initial pH of the concentrate and sorbent type. The systems using CHs-ECH as the ion carrier were significantly more susceptible to the concentrate’s pH changes than these using CHs (Fig. S4. G-L). For example, in the experimental series with CHs-ECH and desorption performed at pH 12, the pH value of the concentrate noted in the last cycles ranged from pH 8.42 to pH 9.48, whereas in the systems with CHs it ranged from pH 11.67 to pH 11.98 (Tab. 2).

Fig. S4. **[A-F] - pH of the source solution (C_start_=10-100 mg P-PO_4_/L) after sorption with CHs and CHs-ECH** **in the successive cycles**: A) CHs, C_start_=10 mg P-PO_4_/L; B) CHs, C_start_=50 mg P-PO_4_/L; C) CHs, C_start_=100 mg P-PO_4_/L; D) CHs-ECH, C_start_=10 mg P-PO_4_/L; E) CHs-ECH, C_start_=50 mg P-PO_4_/L; and F) CHs-ECH, C_start_=100 mg P-PO_4_/L. **[G-I] – pH of the concentrate after desorption in the successive cycles:** G) CHs, C_start_=10 mg P-PO_4_/L; H) CHs, C_start_=50 mg P-PO_4_/L; I) CHs, C_start_=100 mg P-PO_4_/L; J) CHs-ECH, C_start_=10 mg P-PO_4_/L; K) CHs-ECH, C_start_=50 mg P-PO_4_/L; and L) CHs-ECH, C_start_=100 mg P-PO_4_/L. (Sorbent dose = 1 g _d.m._/L, sorption in pH 4 for CHs and pH 3 for CHs-ECH, desorption at pH 12 and 13, Source solution volume to concentrate volume ratio – 5:1, 150 r.p.m., temp. 22 ^o^C).

The increase in source solutions’ pH after sorption in the successive cycles could be due to increasing amounts of sodium cations transferred from the concentrate to the solution. The higher pH values of the source solutions noted in the experimental series with desorption conducted at pH 13 resulted from a higher Na+ concentration in the concentrates. The orthophosphate concentration increasing in the source solutions was accompanied by an increasing concentration of NaOH necessary for concentrate’s pH correction. For this reason, the experimental series with the highest initial concentrations of the source solutions were characterized by the highest pH increase after sorption in the successive cycles (Fig. S4. A-F). The lower pH values of the source solutions in the experimental series with CHs-ECH, compared to these with CHs, were due to the lower sorption pH and the lower pH_PZC_ of the sorbent. Due to, among others, these reasons, the systems with CHs-ECH were characterized by greater changes in concentrates’ pH than the systems with CHs (Fig. S4. G-L).

The greater concentrate’s pH changes in the series with higher initial concentrations of the source solutions were due to, i.a. higher amounts of orthophosphate and chloride anions transferred to the concentrate which decreased solution’s pH. The increased concentrate’s pH after desorption in the successive cycles resulted from decreasing amounts of anions brought to the desorption solution that decreased its pH. Because the final pH of the desorption solution depended on desorption intensity, the experimental series with higher concentrations of the source solutions were characterized by generally lower concentrate’s pH after desorption. The higher efficiency of anion desorption to the concentrate in the successive cycles was also one of the reasons for greater concentrate’s pH changes in the systems with CHs-ECH than in these with CHs (Fig. S4. G-L).
